# Assessing neutrophil-derived ROS production at the bedside: a potential prognostic tool in severe COVID-19 cases

**DOI:** 10.1186/s40635-023-00554-y

**Published:** 2023-10-06

**Authors:** Nazlıhan Boyacı Dündar, David Sarphie, Kenan Yüce, Ümmügülsüm Gaygısız, O. Tolga Kaskatı, Melda Türkoğlu, Gülbin Aygencel Bıkmaz, Lale Karabıyık, Kayhan Çağlar, Gülendam Bozdayı, Rubina Mian, Paul Moss, Mustafa Necmi İlhan

**Affiliations:** 1 Department of Internal Medicine, Division of Intensive Care Medicine, Faculty of Medicine, Gazi University, Ankara, Turkey; 2 Seroxo Limited, Oxford, UK; 3 Department of Clinical Microbiology, Division of Virology, Faculty of Medicine, Gazi University, Ankara, Turkey; 4 Department of Anesthesiology and Reanimation, Division of Intensive Care Medicine, Faculty of Medicine, Gazi University, Ankara, Turkey; 5 Department of Biostatistics, Faculty of Medicine, Lokman Hekim University, Ankara, Turkey; 6 Institute of Immunology and Immunotherapy, University of Birmingham, Birmingham, UK; 7 University Hospitals Birmingham NHS Foundation Trust, Birmingham, UK; 8 Department of Public Health, Faculty of Medicine, Gazi University, Ankara, Turkey

**Keywords:** Neutrophil, COVID-19, Reactive oxygen species (ROS), Leukocyte ImmunoTest

## Abstract

**Purpose:**

A prompt and effective immune response is required for clearance of pathogens but exaggerated states of inflammation can cause extensive collateral damage to the host. We have previously used a rapid near-patient assay that measures the functional capacity of neutrophils to produce reactive oxygen species (ROS) to show that values are elevated in patients with severe COVID-19 or sepsis. Here, we assess the utility of longitudinal ROS measurements to monitor and predict mortality outcome for patients with COVID-19 infection being treated in an ICU setting.

**Methods:**

We used the Leukocyte ImmunoTest™ (LIT™) to quantify neutrophil ROS release using a small volume (10 µL) of capillary blood in a portable, rapid (10-min) format.

**Results:**

ROS values (LIT score) and ROS levels assessed in relation to neutrophil count (LIT/N) were both markedly elevated in the patient group. Furthermore, these correlated strongly with peripheral neutrophil count and CRP value. Serial measurement of neutrophil or CRP values were not able to reliably predict mortality within the study. In contrast, LIT and LIT/N values started to decline at 7 and 5 days, respectively, in patients who survived ICU admission and this increment increased further thereafter.

**Conclusions:**

This study raises the possibility of LIT and LIT/N to be used as a predictive clinical tool for patients with severe COVID-19 and argues for its assessment to inform on prognosis, and potentially guide treatment pathways, in other disorders associated with neutrophil activation.

**Take-home message:**

A longitudinal study of 44 severe COVID-19 patients in the ICU of a leading teaching hospital has demonstrated the prognostic potential of a rapid bedside assay of neutrophil-derived reactive oxygen species (ROS). Assessment of changes in ROS production, as measured using the Leukocyte ImmunoTest^™^, shows that ROS production generally declined back to normal levels for patients who survived, but remained elevated for those patients who did not survive.

The ongoing COVID-19 pandemic has resulted in significant illness and mortality globally, with factors that define disease risk not yet fully understood [1]. Early markers of systemic inflammation, such as serum TNF-α, have shown strong predictive power for clinical outcome; however, their slow kinetics and variability call for the development of faster and more reliable bedside prognostic assays [2].

Although conventional biomarkers, such as C-reactive protein (CRP), neutrophil count, and ferritin, have been useful in predicting clinical outcome, their poor responsiveness and long half-life limit their physiological relevance [3–5]. More dynamic and responsive biomarkers are needed to accurately assess systemic inflammation, as clinical measurements, such as respiratory rate and oxygen saturation, provide limited insights into multi-system inflammatory processes [6].

Recent studies have emphasised the crucial role of neutrophils in the immune dysregulation and hyper-inflammatory response that characterises severe COVID-19 [7–9]. The capacity of neutrophils to produce reactive oxygen species (ROS) can provide valuable insight into the balance between immune activation and tissue damage in COVID-19 patients [9]. At low levels, ROS play an essential role in regulating immune cell function, including neutrophil chemotaxis, phagocytosis, and antigen presentation [10–13]. However, excessive ROS production can lead to tissue damage and organ dysfunction [14, 15].

A key advantage of the LIT assay used here to quantify ROS production is that neutrophils maintain their three-dimensional conformation when ROS production is measured. This conformational preservation closely mimics physiological conditions, providing an accurate representation of cellular behaviour, and maintains G protein-coupled receptors (GPCRs) structures essential for facilitating the assembly and activation of the NADPH oxidase complex which plays a pivotal role in ROS production [16].

In COVID-19 the activation state of neutrophils is a critical determinant of disease progression and a rapid assessment of their activation status is likely to be of importance in determining ultimate clinical outcome [9]. However, the usefulness of neutrophil production of ROS is limited by time-consuming assays that require specialised equipment, and cell separation, purification, and centrifugation can affect their function [17].

Recently a functional assay that assesses the capacity of leukocytes (primarily neutrophils) to produce ROS in response to in vitro chemical stimulation has been demonstrated [9]. The Leukocyte ImmunoTest^™^ (LIT^™^) specifically and rapidly (10 min) quantifies neutrophil ROS release using a small volume of blood obtainable from capillary, arterial or venous sources. As such the LIT score provides a physiologically relevant means for monitoring the cellular capacity of neutrophils to produce superoxide radicals in real time. LIT also avoids centrifugation and ROS production can be studied under near physiological conditions that maintains cells in their 3D structure, enabling engagement with mediators in their immediate environment [18, 19].

In this study, we evaluated the prognostic value of measuring neutrophil-derived ROS production in patients receiving intensive care therapy for COVID-19. The test offers a definitive advantage over traditional laboratory markers in speed and ease of use, requiring only 10 µL of blood and providing bedside results within 10 min. Our results suggest that the LIT and LIT/N values produced by the test are valuable prognostic markers for clinical outcome and have distinct advantages over current laboratory-based assays.

## Methods

### Population

The study (registered at *ClinicalTrials.gov*: NCT05520918) was approved by the Clinical Research Ethics Committee of Gazi University and conducted in the Intensive Care Unit (ICU) of Gazi University Hospital in accordance with the principles of the Declaration of Helsinki, including obtaining informed consent. The patient population consisted of 44 patients who were admitted to the ICU due to COVID-19 pneumonia; the healthy volunteer population consisted of healthcare workers from the hospital. Analysis was performed on clinical samples or via finger-prick assay from healthy volunteers. ROS production in response to phorbol 12-myristate 13-acetate (PMA) stimulation was analysed within 30 min of collection. Diagnosis of COVID status was confirmed using PCR test. Together with a range of conventional haematological tests and clinical observations, the LIT test was performed every day or every other day (except on weekends) until death or discharge from the ICU.

### Data collection

Patients were followed from their admission to ICU up to the day of ICU discharge or death. All data were prospectively collected on standardized study forms. Data variables collected on admission included the demographic characteristics, diagnosis, comorbidities, date of PCR test positivity, symptoms of COVID-19, severity of COVID (according to WHO classification), type of treatment, use of ventilation and ventilation type, PaO_2_/FiO_2_ ratio, use of antibiotics, C-reactive protein (CRP) level, neutrophil count, and total leukocyte count. On admission, disease severity of the patient was evaluated with APACHE II and sequential organ failure assessment (SOFA) score. During ICU stay, patients were assessed for the development of bacterial infection. The vital signs on the days LIT tests were conducted were also recorded. The intensive care unit outcome parameters such as duration of mechanical ventilation, length of ICU stay, length of hospitalization and mortality were also evaluated. The CRP, neutrophil count and total leukocyte count were performed on the day LIT test was conducted on the healthy volunteers.

### Measurements of ROS production by luminometer

We used the Leukocyte ImmunoTest^™^ (LIT^™^, Seroxo, Oxford, UK), a near-patient testing assay for reactive oxygen species (ROS) production in small blood volumes, as a marker of functional neutrophil activation [20–22]. ROS production was measured according to the method previously described [20, 22]. Briefly, a 10 μL sample of freshly obtained blood (obtained by finger prick or venepuncture) was added to 100 µL phosphate buffered saline (PBS) containing phorbol 12-myristate 13-acetate (PMA; Sigma) and luminol. The solution was incubated for 10 min at 37.5 °C. Chemiluminescence was quantified after 10 min using a 3 M handheld luminometer (Clean-Trace, NG3) in relative light units (RLUs). Patients were sampled for LIT measurements regularly at intervals of 2 or 3 days, whilst healthy volunteers were sampled once.

### Statistics

Due to the longitudinal study design, we used linear mixed-effects models, where replicates are nested within subjects, to estimate differences in clinical parameters (PMNL, CRP, LIT, LIT/N) as a function of days from admission for both COVID-19 survivors and non-survivors. These models enable the assessment of the degree of dependency among observations for the same subject.

For nominal variables, boxplots show the distribution of scores by subgroup, and an independent *t* test is used to test differences in LIT means between two independent groups. A Mann–Whitney *U* test is used when the assumptions of the independent *t* test are not met. For ordinal or numerical variables, Spearman correlation coefficients have been computed, including confidence intervals and a test against the null hypothesis that the correlation is zero/the samples come from the same population.

Continuous (quantitative) variables are described as mean ± standard deviation (SD) when showing normal distribution. For variables not showing normal distribution, median and interquartile ranges (IQR) are provided. The categorical (qualitative) variables are described as counts (percentages) and have been analysed with *χ*^2^ (Chi-squared) and Fisher exact tests when necessary.

## Results

### The characteristics of patients with severe COVID-19

Demographic characteristics of the study patients including COVID-19-related symptoms, comorbidities, and ICU course-related therapy and organ supports are shown in Table 1.

**Table 1:** Characteristics of COVID-19 patients (demographics, COVID-19-related symptoms, comorbidities, and ICU course-related therapy and organ supports)

	Survivors (*n* = 24)	Non-survivors (*n* = 20)	*p* value
Demographics			
Gender (%)			0.512
Female	12 (50%)	12 (60%)	
Male	12 (50%)	8 (40%)	
Age (mean ± SD)	59 ± 13	62 ± 11	0.419
APACHE-II score (median; IQR)	10 (6–16)	17 (11–23)	**0.020**
SOFA score (median; IQR)	4 (3–5)	5 (4–8)	**0.003**
COVID-19 symptoms on admission (%)			
Fever	7 (29.2)	8 (40)	0.532
Dizziness	0 (0)	1 (5)	0.268
Headache	3 (12.5)	0 (0)	0.101
Nausea	2 (8.3)	1 (5)	0.662
Coughing	16 (66.7)	15 (75)	0.551
Loss of smell or taste	3 (12.5)	3 (15)	0.810
Abdominal pain	1 (4.2)	0 (0)	0.356
Joint pain	1 (4.2)	1 (5)	0.895
Muscle pain	6 (25)	2 (10)	0.199
Palpitation	1 (4.2)	0 (0)	0.356
Dyspnoea	21 (87.5)	19 (95)	0.394
Chest pain	1 (4.2)	1 (5)	0.895
Confusion	2 (8.3)	2 (10)	0.848
Fatigue	18 (75)	16 (80)	0.697
Sputum	2 (8.3)	7 (35)	0.029
Comorbidities (%)			
Diabetes mellitus	10 (41.7)	10 (50)	0.651
Cardiovascular disease	12 (50)	17 (85)	**0.015**
Respiratory disease	6 (25)	3 (15)	0.376
Neoplasm	3 (12.5)	1 (5)	0.368
Steroid therapy within the last 7 days (%)	12 (50)	12 (60)	0.512
Respiratory support on admission (%)			
Oxygen mask	14 (58.3)	4 (20)	0.63
NIMV	6 (25)	5 (25)	0.545
HFNO	10 (41.7)	10 (50)	0.585
IMV	0 (0)	2 (10)	0.113
Blood gas interpretation on admission (%)			
Respiratory alkalosis	13 (54.2)	12 (60)	0.701
Respiratory acidosis	4 (16.7)	1 (5)	0.225
Metabolic acidosis	1 (4.2)	5 (25)	**0.045**
Renal impairment on admission (%)	0 (0)	5 (25)	**0.09**
Bacterial infection on admission (%)	4 (16.7)	2 (10)	0.521
Baseline laboratory findings on admission (median; IQR)			
Hb (g/dL)	13.4 (12.5–14.0)	11.8 (9.5–13.0)	**0.010**
WBC (× 10^3^/µL)	8.885 (6.530–9.845)	10.470 (6.810–14.385)	0.110
PMNL (× 10^3^/µL)	7.585 (5.165–8.545)	9.720 (5.955–13.545)	**0.037**
% of PMNL	84.65 (81.35–91.20)	92.55 (87.4–94.85)	**0.005**
Lymphocyte (× 10^3^/µL)	0.710 (0.525–0.915)	0.465 (0.355–0.800)	0.179
% of Lymphocyte	7.6 (5.9–11.6)	5.3 (3.0–7.45)	**0.038**
PLT (× 10^3^/µL)	242.5 (183–272.5)	162.5 (150–255)	0.758
d-dimer (μg/mL)	0.78 (0.49–2.07)	1.17 (0,85–1.9)	0.354
Fibrinogen (mg/dL)	554 (431–691)	607 (543–688)	0.724
Sedimentation (mm/h)	81 (36–88)	76 (48–79)	0.946
CRP (mg/L)	80.2 (31.9–177)	130.5 (100.8–197.5)	0.411
PCT (ng/mL)	0.16 (0.07–0.52)	0.49 (0.11–1.75)	0.120
Ferritin (ng/mL)	404 (208–1150)	485 (249–1621)	0.403
IL-6 (pg/mL)	29.72 (9.47–69.13)	93.5 (44.6–186)	**0.029**
LDH (U/L)	369 (306–535)	488 (369–684)	0.156
Trop (ng/L)	7 (5–18)	18 (9–30)	0.300
ALT (U/L)	29 (19–50)	29 (17–33)	0.151
Creatinine (mg/dL)	0.91 (0.65–1.07)	1.04 (0.74–2.25)	0.107
T.Bil (mg/dL)	0.56 (0.42–0.82)	0.59 (0.42–0.82)	0.901
Vital parameters on admission			
Respiratory Rate/min	28 (4–29)	31 (28–36)	0.021
Heart Rate/min	86 (80–11)	93 (85–100)	0.756
Systolic Blood Pressure (mmHg)	128 (115–144)	126 (117–140)	0.848
Diastolic Blood Pressure (mmHg)	73 (63–80)	76 (66–80)	0.313
FiO_2_ on admission (%)	60 (45–80)	90 (78–100)	**0.000**
ICU course-related factors (%)			
Requirement of RRT	0 (0)	7 (35)	**0.020**
Requirement of IMV	1 (4.2)	20 (100)	**0.000**
Requirement of vasopressor	0 (0)	17 (85)	**0.000**
Newly diagnosed bacterial infection	0 (0)	9 (45)	**0.000**
Pulse steroid (%)	12 (50)	11 (55)	0.744
Anti-inflammatory therapy (%)	4 (16.7)	7 (35)	0.167
LOS for ICU (median; IQR)	6 (4–8)	11 (7–22)	**0.003**

*ALT* alanine aminotransferase, *APACHE* acute physiology and chronic health evaluation, *COVID-19* coronavirus disease 2019, *CRP* c-reactive protein, *FiO*_*2*_ fraction of inspired oxygen, *HFNO* high flow nasal cannula oxygen therapy, *ICU* intensive care unit, *IL-6* interleukin-6, *IMV* invasive mechanical ventilation, *LDH* lactate dehydrogenase, *LOS* length of stay, *NIMV* non-invasive mechanical ventilation, *PCT* procalcitonin, *PLT* platelet count, *PMNL* polymorphonuclear leukocytes, *RRT* renal replacement therapy, *SOFA* sequential organ failure score, *T.Bil* total bilirubin, *Trop* troponin, *WBC* white blood cell count

### ROS production in peripheral blood is increased in patients with severe COVID-19

**Fig. 1:**
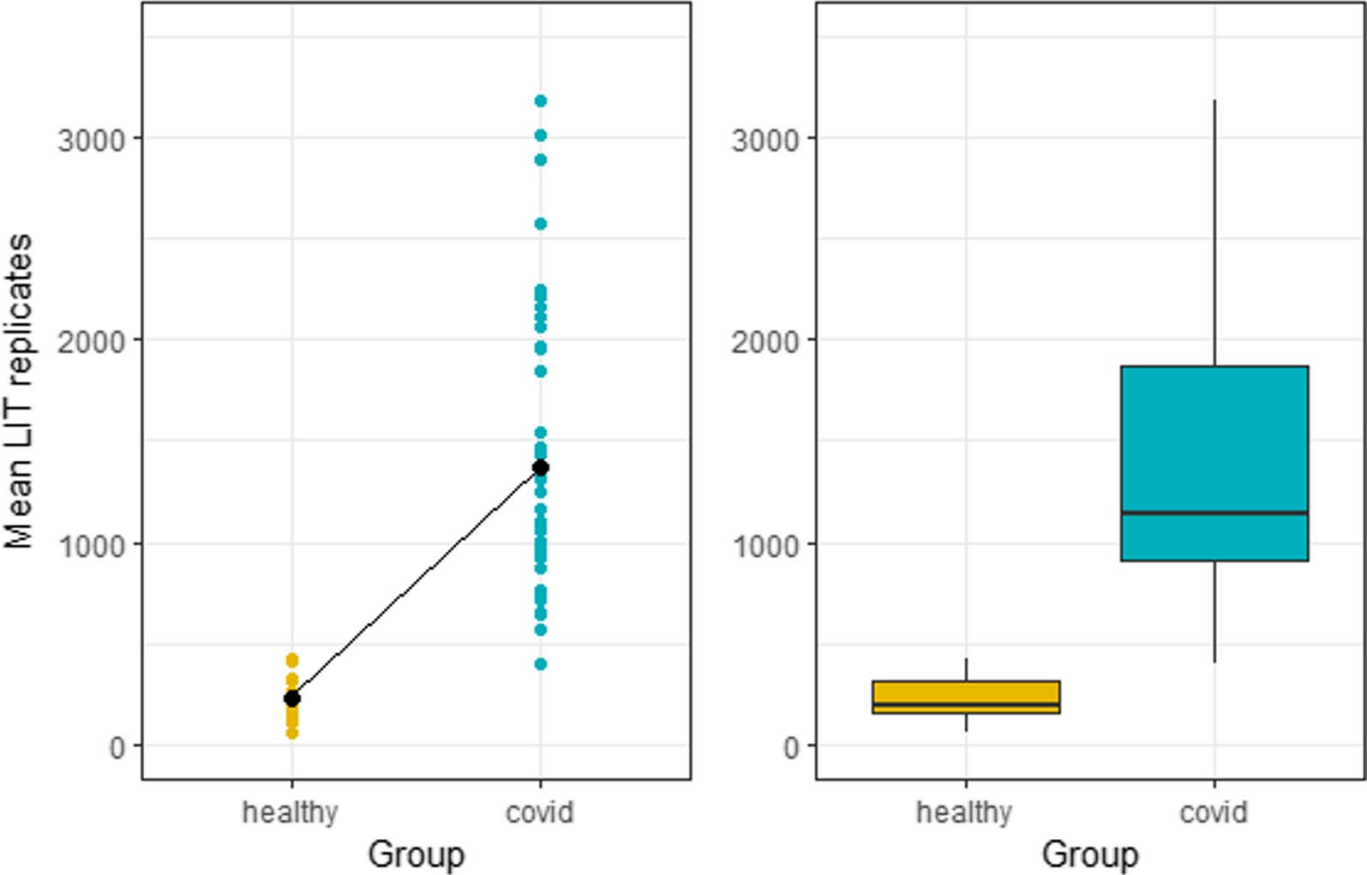
Average LIT™ scores are higher in COVID-19 patients compared to healthy volunteers. Absolute LIT scores in healthy volunteer group (single replicate) and COVID patient group (up to ten longitudinal replicates) are shown on the left as individual subject values and mean for the group shown as thicker dot. The boxplot on the right-hand side shows the median and interquartile range of the scores for both groups

Near-patient testing to determine the intensity of ROS production (LIT score) was undertaken at the time of ICU admission for treatment of COVID-19 and at serial timepoints thereafter. Data comparing all LIT scores from patients and healthy cohorts are presented in Fig. 1. Median LIT score for patients with COVID-19 was 1135 (IQR: 910–1871) and was 5.8-fold higher than values within the control group (median: 196; IQR: 160–310, Mann–Whitney *U* test, *p* <  <  < 0.05).

**Fig. 2:**
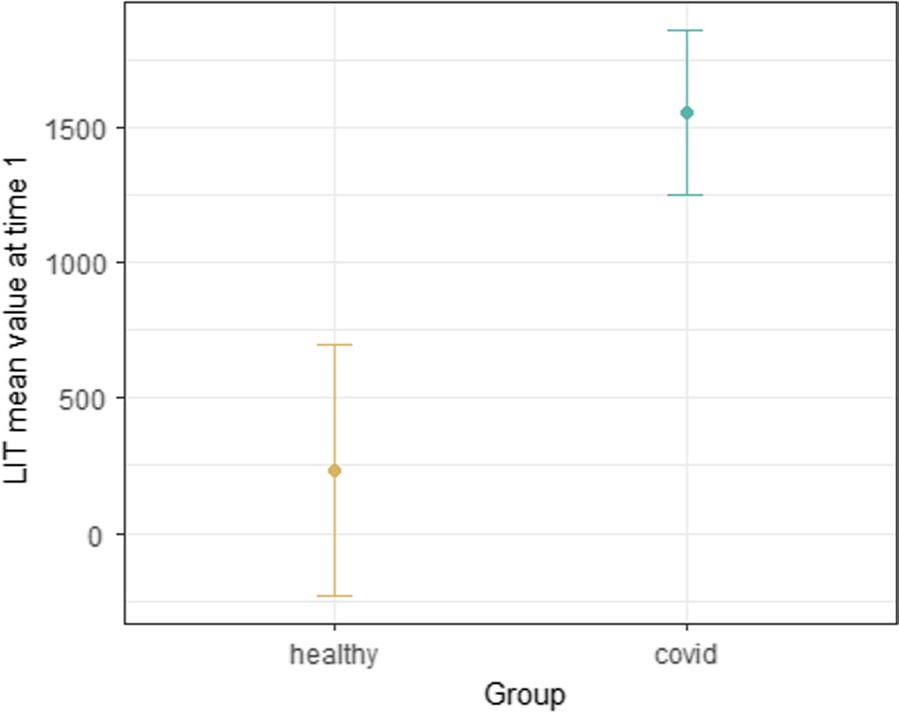
Predicted marginal LIT means for COVID patients are higher compared with those for healthy volunteers at baseline. Results of linear regression analysis of LIT results at baseline (timepoint 1) giving predicted marginal LIT mean of 1557 for COVID patients compared with 234 for healthy subjects

We conducted a linear regression analysis of LIT scores at baseline to determine whether there was a significant difference in baseline scores between patients and healthy subjects. Results showed significantly higher values in the patient group (*p* < 0.01), with a predicted marginal LIT mean of 1557 for patients and 234 for healthy subjects (Fig. 2). These results demonstrate that the baseline and aggregate LIT score is markedly elevated in ICU patients with COVID-19 compared to healthy volunteers.

Results of linear regression analysis of LIT results at baseline (timepoint 1) giving predicted marginal LIT mean of 1557 for COVID patients compared with 234 for healthy subjects.

### ROS production is strongly correlated with neutrophil cell count and CRP

**Fig. 3:**
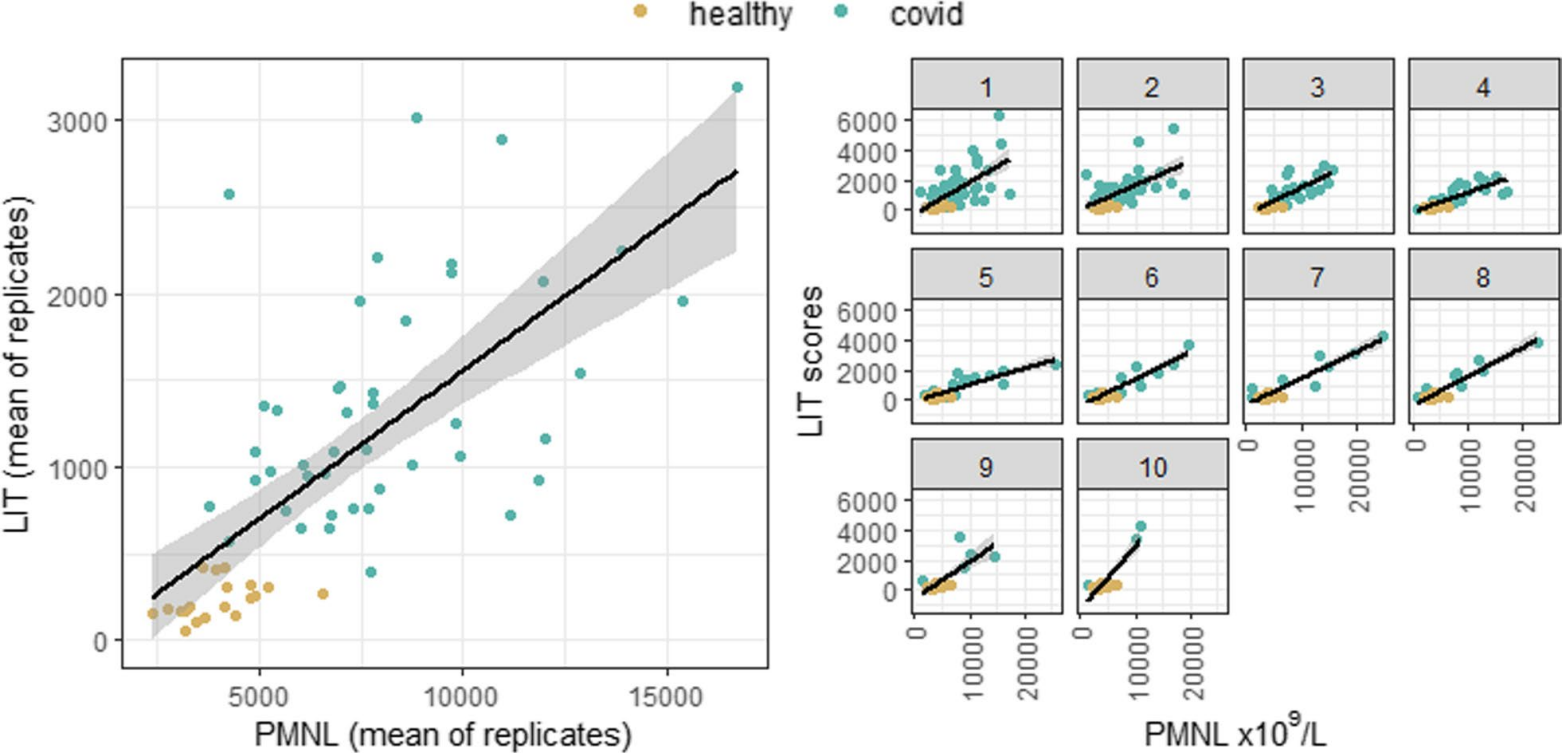
Association of LIT and PMNL per group: mean of replicates and over time. These results demonstrate a high positive correlation between LIT scores (mean of replicates) and PMNL measurements for both patients (green) and healthy volunteers (gold), echoing results from the previous study of LIT in COVID and sepsis patients^9^. Individual graphs on the right show association between LIT scores and PMNL measurements at specific timepoints during patients’ ICU course (up to 10 LIT readings for some patients.) In each case, samples for LIT readings and samples for PMNL measurements were taken concurrently

We then compared LIT values with matched neutrophil count values (PMNL) within the patient and healthy groups (Fig. 3). Neutrophil count is a key haematological parameter and is used widely in clinical practice. LIT values were found to correlate strongly with PMNL values and support the assessment of the LIT score as a measure of the functional capacity of neutrophils within blood (Spearman’s correlation 0.75 (0.59–0.86); *p* < 0.05).

These results demonstrate a high positive correlation between LIT scores (mean of replicates) and PMNL measurements for both patients (green) and healthy volunteers (gold), echoing results from the previous study of LIT in COVID and sepsis patients^9^. Individual graphs on the right show association between LIT scores and PMNL measurements at specific timepoints during patients’ ICU course (up to 10 LIT readings for some patients.) In each case, samples for LIT readings and samples for PMNL measurements were taken concurrently.

**Fig. 4:**
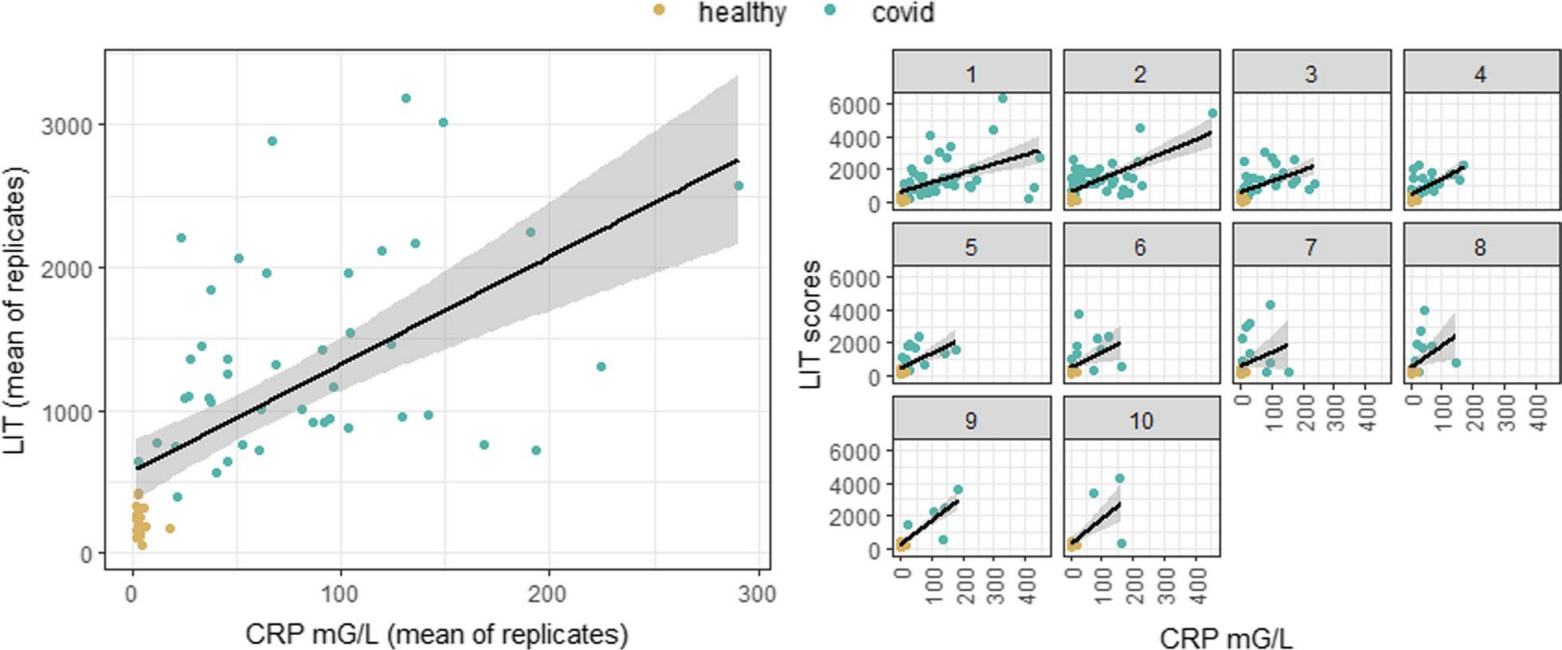
Association of LIT and CRP mg/L per group: mean of replicates and over time. These results demonstrate a high correlation between LIT (mean of replicates) and CRP mG/L (mean of replicates). Again, the individual graphs on the right show association between LIT scores and CRP measurements at specific timepoints during patients’ ICU course (up to 10 LIT readings for some patients.) As above, samples for LIT readings and samples for CRP measurements were taken concurrently

The potential association between LIT and the inflammatory marker C-reactive protein (CRP) was also determined and found to be strongly correlated (Spearman’s correlation coefficient 0.73 (0.58–0.83), *p* < 0.05 (Fig. 4).

These results demonstrate a high correlation between LIT (mean of replicates) and CRP mG/L (mean of replicates). Again, the individual graphs on the right show association between LIT scores and CRP measurements at specific timepoints during patients’ ICU course (up to 10 LIT readings for some patients.) As above, samples for LIT readings and samples for CRP measurements were taken concurrently.

### Sustained high LIT values are observed in patients who succumb to infection

**Fig. 5:**
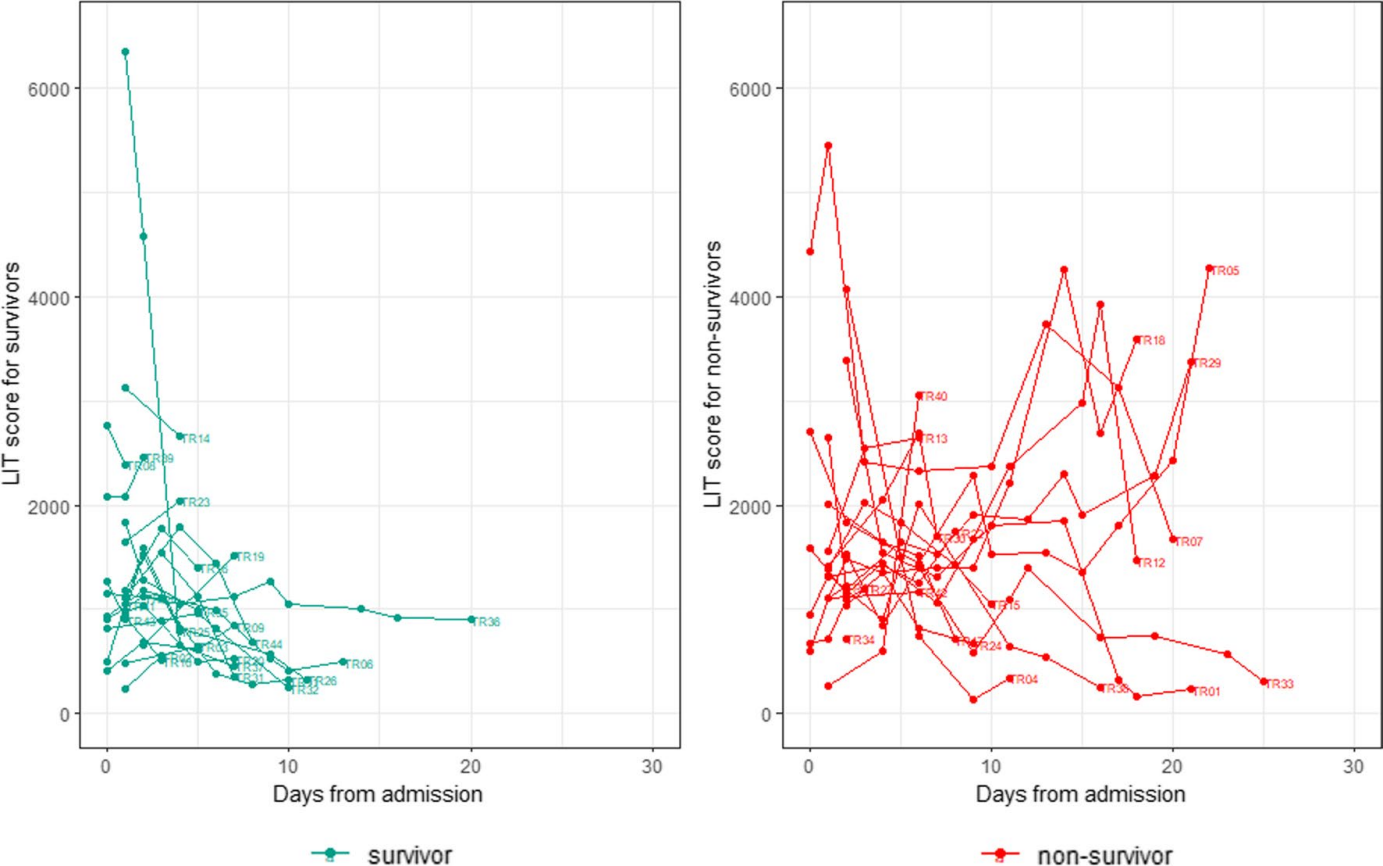
Trajectories of LIT scores vs days from admission for survivors (left) and non-survivors (right). These graphs highlight the distinct difference in trajectories of LIT scores between patients with different outcomes, specifically survivors and non-survivors. In particular, non-survivors generally maintained higher LIT scores (> 1000 RLUs) for longer periods compared with survivors

Next we compared the trajectory of LIT scores for individual patients and related this to survival (Fig. 5). A distinct difference was observed in the profile of LIT scores with high LIT scores of > 1000 RLUs maintained for longer periods in patients who succumbed to COVID-19.

These graphs highlight the distinct difference in trajectories of LIT scores between patients with different outcomes, specifically survivors and non-survivors. In particular, non-survivors generally maintained higher LIT scores (> 1000 RLUs) for longer periods compared with survivors.

### Distribution of LIT/N per group: healthy subjects and COVID patients

**Fig. 6:**
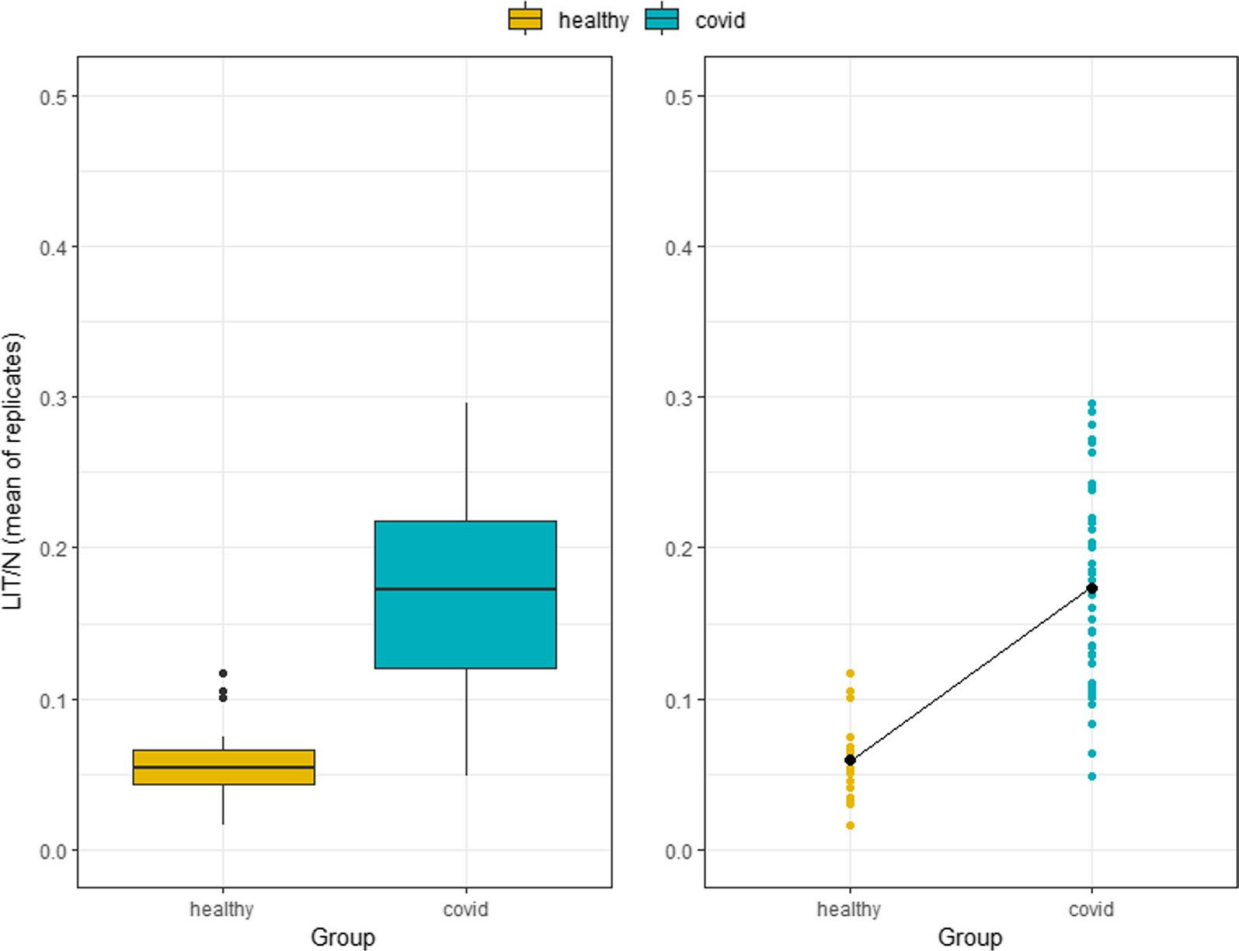
LIT/N score by COVID and volunteer group (boxplot and dot plot with mean). The boxplot on the left-hand side shows the median and interquartile range of the scores for both groups (healthy and COVID), while the dot plot on the right-hand side shows individual subject values and, as a thicker dot, the mean for the group. The *y*-axis shows the mean value over the technical replicates of the LIT/N measurements (between 1 and 10)

A previous study has highlighted the potential importance of using LIT score relative to neutrophil count (LIT/N) as a new parameter for monitoring clinical progression in COVID-19 patients [9]. As such, the LIT/N values within the cohort were determined in 44 COVID patients and 19 healthy volunteers. Median LIT/N values for COVID patients exceeded those in healthy donors (0.172 vs 0.054) and the range of values was also higher in the patient group (COVID IQR: 0.121–0.218 vs Healthy IQR: 0.043–0.066) (Fig. 6 and Table 2).

**Table 2:** Mean (SD) and median (IQR) LIT/N values for COVID and volunteer groups

Group	*N*	Mean ± SD	Median (IQR)
Healthy	19	0.059 ± 0.026	0.054 (0.043–0.066)
COVID	44	0.174 ± 0.065	0.172 (0.121–0.218)

### Linear mixed effects model for COVID non-survivors and survivors: PMNL, CRP, LIT and number of days from admission

We then ran a linear mixed effects model on the patient group to account for the replicates within each subject with the dependent variable taking as values PMNL, CRP and LIT as a function of number of days from ICU admission. Healthy volunteers were excluded due to the relatively constant LIT values within this cohort. Results are highlighted below for each parameter:

#### PMNL

**Fig. 7:**
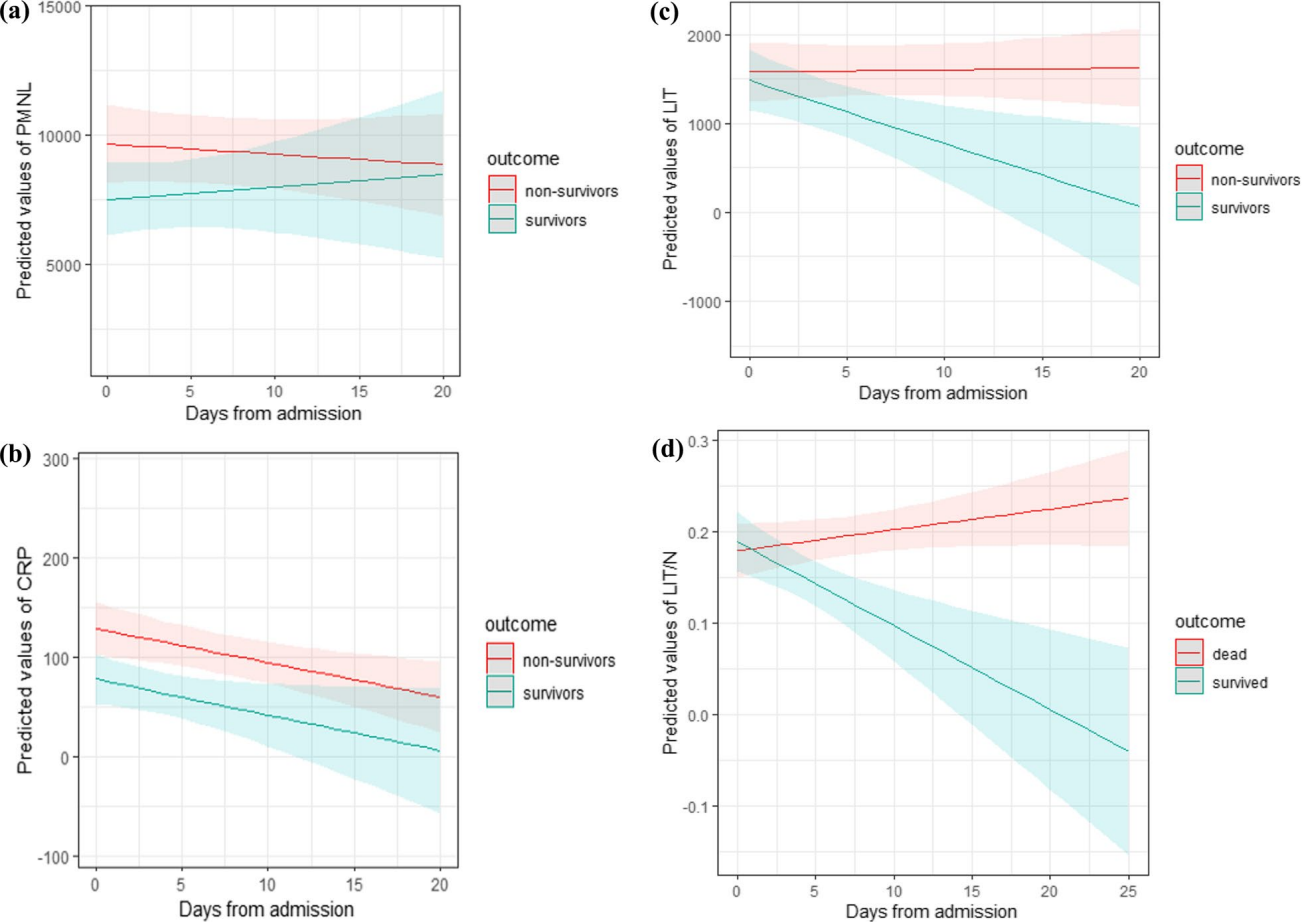
**a** Linear mixed effect model using neutrophils (PMNL) only. This interaction plot of predicted values of PMNL per days from admission, per outcome (survivors, non-survivors) shows that PMNL values (neutrophil counts alone) are not different between survivors and non-survivors when varying the number of days from admission. **b** Linear mixed effect model using C-reactive protein (CRP). This interaction plot gives predicted values of CRP per days from admission, per outcome (survivors, non-survivors). This graph shows that the CRP measurements for interaction between days from admission and outcome both trend downward at approximately the same rates and are not significantly different for the two outcomes. **c** Linear mixed effect model using LIT. This interaction plot of predicted values of LIT per days from admission, per outcome shows that the predicted LIT values decrease as time progresses for survivors as opposed to non-survivors. **d** Linear mixed effect model using LIT/N. This interaction plot of predicted values of LIT/N per days from admission, per outcome shows that as the number of days from admission increases, predicted LIT/N values between survivors and non-survivors diverge. Survivors have reducing values of LIT/N as time progresses, whereas non-survivors present an increase as time progresses

Figure 7 shows that PMNL values are not significantly different between survivors and non-survivors when varying the number of days from admission and that values of PMNL between survivors and non-survivors do not change significantly when the number of days from admission increases.

#### CRP

Figure 7 shows results of linear mixed effect model using C-reactive protein (CRP) giving predicted values of CRP per days from admission, per outcome (survivors, non-survivors). This graph shows that the CRP measurements for interaction between days from admission and outcome both trend downward at approximately the same rates and are not significantly different for the two outcomes.

#### LIT

Figure 7 shows an interaction plot of predicted values of LIT per days from admission, per outcome for survivors and non-survivors. This graph shows that the predicted LIT values decrease as time progresses for survivors as opposed to non-survivors.

### Linear mixed effects model in relation to COVID-19 mortality: LIT/N and number of days from admission

LIT/N values showed a trend to a progressive decrease for patients who survived compared to those who did not. The predicted marginal mean value for LIT/N was 0.19 in patients who did not survive whilst estimated at 0.13 for survivors at 6.44 days, which was the average number of days from admission (Fig. 7). The results of this model suggest that as the number of days from admission increases, predicted LIT/N values between survivors and non-survivors diverge. As such the model suggests that patients who survive COVID-19 exhibit a gradual reduction in LIT/N over time, whilst this is not seen in non-survivors. This divergence was not seen with measurement of PMNL or CRP and indicates that LIT/N may represent an important prognostic parameter in this setting.

## Discussion

It is well-established that neutrophils can act as important mediators of immunopathology and it has been previously demonstrated that ROS production is elevated in patients with COVID-19 [9]. Here, we sought to assess the prognostic impact of ROS measurement in relation to mortality in a cohort of patients admitted to ICU with COVID-19.

Our study confirmed previous work that COVID-19 values at entry to ICU are markedly elevated compared to healthy donors and reflect the high levels of peripheral ROS production in patients with pulmonary complications of COVID-19 [9]. The reasons for increased ROS production in COVID patients are likely to be multi-factorial and may relate to the immature or dysfunctional mature neutrophil phenotype reported in severe COVID-19 or direct activation of neutrophils through ACE2 engagement. It is uncertain if ROS is a direct mediator of tissue damage but neutrophil infiltration is thought to contribute to pulmonary complications and ROS production initiates a range of downstream activities, including generation of neutrophil extracellular traps (NETs) and stimulation of production of pro-inflammatory cytokines [18, 23–25].

As expected, LIT values were strongly correlated with the peripheral neutrophil count, as this population is the dominant source of ROS and this relationship has been reported previously [9]. However, we also observe a strong association with CRP values, an important systemic marker of inflammation which is synthesized in the liver. As such, the degree of ROS production is directly related to the overall inflammatory profile but whether ROS acts as a causative factor, or a simple correlate of the inflammatory state, is currently unknown.

The major aim of our study was to assess the utility of the near-patient LIT assay for ROS production as a predictive biomarker for clinical outcome in patients with COVID-19. In particular, within the study cohort of 44 patients, 24 (54.5%) survived the ICU admission and were discharged into ward care, whilst 20 patients (45.5%) died whilst on ICU (mortality timepoint cutoff: 42 days).

We initially determined the prospective neutrophil counts over time within these two groups. As expected from prior literature [18], neutrophil counts were somewhat higher within the mortality cohort, but the confidence interval overlapped, so that this was not an effective predictive marker. Elevated CRP values are also seen in patients with severe COVID-19 and a trend towards increased values within the mortality cohort was seen between days 1 and 10 of ICU admission, although the predictive value was not sustained.

LIT values had better prognostic value and diverged consistently, and with increasing magnitude, between the two study groups (survivors and non-survivors) beyond day 7 of infection. As such, the measurement of LIT values may represent an important prognostic marker for this disorder. An even more noticeable effect was seen in relation to LIT/N values which diverged markedly after day 5 of ICU admission, falling substantially within the group that survived, and with an effect that increased over time. These findings indicate that LIT/N measurement may represent a novel and highly predictive marker of clinical outcome for patients undergoing ICU care for COVID-19. Importantly, the LIT test has several technical advantages over current tests as it is a near-patient assay, delivers a result within 10 min from blood droplets from venepuncture samples or finger prick assays, and is inexpensive.

The statistical validity of this approach would need to be assessed in larger clinical studies, although this will be challenging to deliver at the current time due to the low rate of ICU admission for COVID-19 patients in the current era of vaccination and high levels of prior infection. As such, the utility of LIT/N should also be assessed in other clinical settings on ICU, such as patients with sepsis, where our initial data have already shown high levels of LIT/N at the time of ICU admission. As the LIT test is portable and inexpensive it can be used in hospitals, outreach clinics and in the field, and raises the additional possibility of widening access of information available to clinicians in isolated regions and in low- and middle-income countries. In particular, if predictive value is confirmed and defined, it could potentially be used to triage patients, thus freeing up vital resources.

Our study has a number of limitations. The cohort size is modest, although the denominator of 44 patients with severe disease is larger than many other prognostic assessments and was undertaken when the incidence of severe disease was falling. The demographic details of the study group are also somewhat limited, and we do not have long-term clinical follow-up data. Finally, ROS production is only one measure of neutrophil activation and additional measurements may serve to increase the predictive power of this phenotype. For example, while this study did not directly investigate the role of neutrophil extracellular traps (NETs), future research may benefit from studying the correlation between ROS generation and NETs.

For decades, the clinical assessment of neutrophils within disease has relied on the quantification of cells within blood, aided in some situations with morphological analysis of a blood smear. Crucially, these fail to assess neutrophil function, and here we use the LIT test and corrected LIT/N values to assess this rapidly at the patient bedside. We show that these values appear highly promising as predictive markers of mortality in a patient group on ICU. Given that neutrophils are known to orchestrate cellular damage in various disease conditions, such as sepsis [15, 26], the LIT/N biomarker has the potential to be a powerful prognostic tool in assessing disease severity in those conditions as well. As such we propose that larger studies are undertaken in conditions, such as sepsis to assess the potential importance of these values to aid prognosis, and potentially direct treatment pathways, in disorders, where neutrophil activation may act as a determinant of disease course.

## Conclusions

This study evaluated the clinical utility of a point-of-care (POC) diagnostic assay for rapid assessment of immune dysregulation as a tool for monitoring patients with severe COVID-19 in an ICU. The LIT assay assesses stimulated production of reactive oxygen species (ROS) which has been shown to be elevated in both COVID and sepsis. The current study found that ROS levels (LIT score) and ROS levels measured relative to neutrophil count (LIT/N) decreased to normal levels in patients who survived but remained elevated in patients who did not survive. Our findings lay the foundations for a new assessment of cellular function as a rapid POC test that may help to monitor disease progression in COVID, sepsis and other inflammation-linked conditions. Furthermore, the LIT assay may also offer the possibility of guiding the introduction of immunomodulatory therapies to optimize innate cell function and improve clinical outcome. This possibility of rapidly guiding bespoke therapeutic strategies represents a paradigm shift in our approach to disease management and treatment.

## Data Availability

The raw data set has been published on figshare at: https://doi.org/10.6084/m9.figshare.22568539.

## Abbreviations

ALT: Alanine aminotransferase
APACHE: Acute physiology and chronic health evaluation
COVID-19: Coronavirus disease 2019
CRP: C-reactive protein
FiO_2_: Fraction of inspired oxygen
HFNO: High-flow nasal cannula oxygen therapy
ICU: Intensive care unit
IL-6: Interleukin-6
IMV: Invasive mechanical ventilation
IQR: Interquartile range
LDH: Lactate dehydrogenase
LIT: Leukocyte ImmunoTest score
LIT/N™: Leukocyte ImmunoTest score normalised by neutrophil count
LOS: Length of stay (ICU)
NIMV: Non-invasive mechanical ventilation
PCT: Procalcitonin
PLT: Platelet count
PMNL: Polymorphonuclear leukocytes (neutrophil count)
RRT: Renal replacement therapy
SOFA: Sequential organ failure score
T.Bil: Total bilirubin
Trop: Troponin
WBC: White blood cell count

## References

[1] Del Rio C, Malani PN (2020). COVID-19-new insights on a rapidly changing epidemic. JAMA.

[2] Del Valle DM, Kim-Schulze S, Huang H-H (2020). An inflammatory cytokine signature predicts COVID-19 severity and survival. Nat Med.

[3] Wang G, Wu C, Zhang Q (2020). C-reactive protein level may predict the risk of COVID-19 aggravation. Open Forum Infect Dis.

[4] Lippi G, Plebani M, Henry BM (2020). Thrombocytopenia is associated with severe coronavirus disease 2019 (COVID-19) infections: a meta-analysis. Clin Chim Acta.

[5] Mehta P, McAuley DF, Brown M, Sanchez E, Tattersall RS, Manson JJ (2020). COVID-19: consider cytokine storm syndromes and immunosuppression. Lancet.

[6] Chen G, Wu D, Guo W (2020). Clinical and immunological features of severe and moderate coronavirus disease 2019. J Clin Invest.

[7] Lucas C, Wong P, Klein J (2020). Longitudinal analyses reveal immunological misfiring in severe COVID-19. Nature.

[8] Mathew D, Giles JR, Baxter AE (2020). Deep immune profiling of COVID-19 patients reveals distinct immunotypes with therapeutic implications. Science.

[9] Veenith T, Martin H, Le Breuilly M (2022). High generation of reactive oxygen species from neutrophils in patients with severe COVID-19. Sci Rep.

[10] Lambeth JD (2004). NOX enzymes and the biology of reactive oxygen. Nat Rev Immunol.

[11] Sies H (2014). Role of metabolic H2O2 generation: redox signaling and oxidative stress. J Biol Chem.

[12] Riganti C, Gazzano E, Polimeni M, Aldieri E, Ghigo D (2012). The pentose phosphate pathway: an antioxidant defense and a crossroad in tumor cell fate. Free Radic Biol Med.

[13] Schieber M, Chandel NS (2014). ROS function in redox signaling and oxidative stress. Curr Biol.

[14] Barnes BJ, Adrover JM, Baxter-Stoltzfus A (2020). Targeting potential drivers of COVID-19: neutrophil extracellular traps. J Exp Med.

[15] Mantovani A, Cassatella MA, Costantini C, Jaillon S (2011). Neutrophils in the activation and regulation of innate and adaptive immunity. Nat Rev Immunol.

[16] Futosi K, Fodor S, Mócsai A (2013). Neutrophil cell surface receptors and their intracellular signal transduction pathways. Int Immunopharmacol.

[17] Winterbourn CC (2008). Reconciling the chemistry and biology of reactive oxygen species. Nat Chem Biol.

[18] McKenna E, Wubben R, Isaza-Correa JM (2022). Neutrophils in COVID-19: not innocent bystanders. Front Immunol.

[19] Nishida N, Xie C, Shimaoka M, Cheng Y, Walz T, Springer TA (2006). Activation of leukocyte beta2 integrins by conversion from bent to extended conformations. Immunity.

[20] Shelton-Rayner GK (2010). Leukocyte reactivity as an objective means of quantifying mental loading during ergonomic evaluation. Cell Immunol.

[21] McLaren GW (2003). Leukocyte coping capacity: a novel technique for measuring the stress response in vertebrates. Exp Physiol.

[22] Fullerton, J. et al (2019) Repurposing the Oxford MediStress Leukocyte Coping CapacityTM assay as a novel point-of-care biomarker of neutrophil function. Presentation British Pharmacological Society Edinburgh, UK

[23] Brinkmann V, Laube B, Abu Abed U, Goosmann C, Zychlinsky A (2010). Neutrophil extracellular traps: how to generate and visualize them. J Vis Exp.

[24] Naik E, Dixit VM (2011). Mitochondrial reactive oxygen species drive proinflammatory cytokine production. J Exp Med.

[25] Sheshachalam A, Srivastava N, Mitchell T, Lacy P, Eitzen G (2014). Granule protein processing and regulated secretion in neutrophils. Front Immunol.

[26] Kolaczkowska E, Kubes P (2013). Neutrophil recruitment and function in health and inflammation. Nat Rev Immunol.

